# Hydroxychloroquine levels in pregnancy and materno-fetal outcomes in systemic lupus erythematosus patients

**DOI:** 10.1093/rheumatology/keae302

**Published:** 2024-06-05

**Authors:** Gelsomina Alle, Gaëlle Guettrot-Imbert, Maddalena Larosa, Anne Murarasu, Estibaliz Lazaro, Nathalie Morel, Pauline Orquevaux, Laurent Sailler, Viviane Queyrel, Eric Hachulla, Françoise Sarrot Reynauld, Laurent Pérard, Alice Bérezné, Chafika Morati-Hafsaoui, Elodie Chauvet, Christophe Richez, Tiphaine Goulenok, Jonathan London, Anna Molto, Geoffrey Urbanski, Maëlle Le Besnerais, Vincent Langlois, Gaëlle Leroux, Odile Souchaud-Debouverie, Céline Lartigau Roussin, Vincent Poindron, Benoit Blanchet, Emmanuelle Pannier, Loïc Sentilhes, Luc Mouthon, Véronique Le Guern, Nathalie Costedoat-Chalumeau, GR2 Study Group

**Affiliations:** Service de Médecine Interne, Centre de référence des maladies auto-immunes et auto-inflammatoires systémiques rares d'Ile-de-France, de l’Est et de l’Ouest, Hôpital Cochin, Assistance Publique-Hôpitaux de Paris (AP-HP), Université Paris Cité, Paris, France; Rheumatology Section, Internal Medicine Service, Hospital Italiano de Buenos Aires, Buenos Aires, Argentina; Service de Médecine Interne, Centre de référence des maladies auto-immunes et auto-inflammatoires systémiques rares d'Ile-de-France, de l’Est et de l’Ouest, Hôpital Cochin, Assistance Publique-Hôpitaux de Paris (AP-HP), Université Paris Cité, Paris, France; Unit of Rheumatology, Department of Medical Specialties, Ospedale La Colletta, Genoa, Italy; Service de Médecine Interne, Centre de référence des maladies auto-immunes et auto-inflammatoires systémiques rares d'Ile-de-France, de l’Est et de l’Ouest, Hôpital Cochin, Assistance Publique-Hôpitaux de Paris (AP-HP), Université Paris Cité, Paris, France; Département de Médecine Interne et Immunologie Clinique, Centre Hospitalier Régional Universitaire de Tours, Tours, France; INSERM, Équipe ECAMO, CRESS (Centre of Research in Epidemiology and StatisticS), UMR 1153, Université Paris-Cité, Paris, France; Service de Médecine Interne, Hôpital du Haut-Lévêque, Centre Hospitalier Universitaire de Bordeaux, Centre de Référence des Maladies Auto-Immunes et auto-Inflammatoires Systémiques Rares de l’Est et du Sud-Ouest, Bordeaux, France; Service de Médecine Interne, Centre de référence des maladies auto-immunes et auto-inflammatoires systémiques rares d'Ile-de-France, de l’Est et de l’Ouest, Hôpital Cochin, Assistance Publique-Hôpitaux de Paris (AP-HP), Université Paris Cité, Paris, France; Service de Médecine Interne, Hôpital Robert Debré, Centre Hospitalier Universitaire de Reims, Reims, France; Service de Médecine Interne, Centre Hospitalier Universitaire de Toulouse, Toulouse, France; Service de Rhumatologie, Centre Hospitalier Universitaire de Nice, Nice, France; Université de Lille, INSERM, Centre Hospitalier Universitaire de Lille, Service de Médecine Interne et Immunologie Clinique, Centre de référence des Maladies Auto-Immunes et Auto-inflammatoires Systémiques rares de l'Adulte du Nord, Nord-Ouest, Méditerranée et Guadeloupe (CeRAINOM), U1286-INFINITE: Institute for Translational Research in Inflammation, Lille, France; Service de Médecine Interne, Centre Hospitalier Universitaire Grenoble Alpes, Grenoble, France; Service de Médecine Interne, Centre Hospitalier Saint Joseph Saint Luc, Lyon, France; Service d’Infectiologie et Médecine Interne, Centre de compétence des maladies auto-immunes et auto-inflammatoires systémiques rares, Centre Hospitalier Annecy-Genevois, Annecy, France; Service d’Infectiologie et Médecine Interne, Centre de compétence des maladies auto-immunes et auto-inflammatoires systémiques rares, Centre Hospitalier Annecy-Genevois, Annecy, France; Service de Médecine Interne, Centre Hospitalier de Perpignan, Perpignan, France; Service de Rhumatologie, Centre Hospitalier Universitaire de Bordeaux, Centre de référence des maladies auto-immunes et auto-inflammatoires systémiques rares de l’Est et du Sud-Ouest, Bordeaux, France; Service de Médecine Interne, AP-HP Hôpital Bichat Claude Bernard, Paris, France; Service de Médecine Interne, Centre de référence des maladies auto-immunes et auto-inflammatoires systémiques rares d'Ile-de-France, de l’Est et de l’Ouest, Hôpital Cochin, Assistance Publique-Hôpitaux de Paris (AP-HP), Université Paris Cité, Paris, France; Service de Rhumatologie, AP-HP Hôpital Cochin, Paris, France; Service de Médecine Interne et d'Immunologie clinique, Centre Hospitalier Universitaire Angers, Angers, France; Service de Médecine Interne, Centre Hospitalier Universitaire Rouen, Rouen, France; Service de Médecine Interne et Maladies Infectieuses, Hôpital Le Havre, Le Havre, France; Service de Médecine Interne, AP-HP Hôpital Pitié-Salpêtrière, Paris, France; Service de Médecine Interne, Centre Hospitalier Universitaire Poitiers, Poitiers, France; Service de Médecine Interne et d'Immunologie clinique, Centre Hospitalier Ouest Réunion, Saint Paul, France; Service d’Immunologie Clinique, Nouvel Hôpital Civil, Strasbourg, France; Biologie du Médicament—Toxicologie, CARPEM, AP-HP Hôpital Cochin—Université Paris Cité, Paris, France; Service d’Obstétrique, Maternité Port-Royal, Hôpital Cochin, Paris, France; Service de Gynécologie Obstétrique, Centre Hospitalier Universitaire de Bordeaux, Bordeaux, France; Service de Médecine Interne, Centre de référence des maladies auto-immunes et auto-inflammatoires systémiques rares d'Ile-de-France, de l’Est et de l’Ouest, Hôpital Cochin, Assistance Publique-Hôpitaux de Paris (AP-HP), Université Paris Cité, Paris, France; Service de Médecine Interne, Centre de référence des maladies auto-immunes et auto-inflammatoires systémiques rares d'Ile-de-France, de l’Est et de l’Ouest, Hôpital Cochin, Assistance Publique-Hôpitaux de Paris (AP-HP), Université Paris Cité, Paris, France; Service de Médecine Interne, Centre de référence des maladies auto-immunes et auto-inflammatoires systémiques rares d'Ile-de-France, de l’Est et de l’Ouest, Hôpital Cochin, Assistance Publique-Hôpitaux de Paris (AP-HP), Université Paris Cité, Paris, France

**Keywords:** systemic lupus erythematosus, pregnancy, hydroxychloroquine, maternal flares, adverse pregnancy outcomes, therapeutic drug monitoring

## Abstract

**Objectives:**

Data about hydroxychloroquine (HCQ) levels during pregnancy are sparse. We assessed HCQ whole-blood levels at first trimester of pregnancy as a potential predictor of maternal and obstetric/fetal outcomes in patients with systemic lupus erythematosus (SLE).

**Methods:**

We included pregnant SLE patients enrolled in the prospective GR2 study receiving HCQ, with at least one available first-trimester whole-blood HCQ assay. We evaluated several cut-offs for HCQ whole-blood levels, including ≤200 ng/ml for severe non-adherence. Primary outcomes were maternal flares during the second and third trimesters of pregnancy, and adverse pregnancy outcomes (APOs: fetal/neonatal death, placental insufficiency with preterm delivery, and small-for-gestational-age neonates).

**Results:**

We included 174 patients (median age: 32.1 years, IQR 28.8–35.2). Thirty (17.2%) patients had flares, four (2.3%) being severe. APOs occurred in 28 patients (16.1%). There were no significant differences in APOs by HCQ level for either those with subtherapeutic HCQ levels (≤500 ng/ml *vs* >500 ng/ml: 23.5% *vs* 14.3%, *P* *=* 0.19) or those with non-adherent HCQ levels (≤200 ng/ml *vs* >200 ng/ml: 20.0% *vs* 15.7%, *P* *=* 0.71). Similarly, the overall rate of maternal flares did not differ significantly by HCQ level cut-off, but patients with subtherapeutic (HCQ ≤500 ng/ml: 8.8% *vs* 0.7%, *P* *=* 0.02) and non-adherent HCQ levels (≤200 ng/ml: 13.3% *vs* 1.3%, *P* *=* 0.04) had significantly more severe flares.

**Conclusion:**

In this large prospective study of pregnant SLE patients, first-trimester subtherapeutic (≤500 ng/ml) and severe non-adherent (≤200 ng/ml) HCQ levels were associated with severe maternal flares, but not with APOs.

**Trial registration:**

ClinicalTrials.gov, http://clinicaltrials.gov, NCT02450396

Rheumatology key messages

- First-trimester subtherapeutic and severe non-adherent HCQ levels were associated with severe maternal flares in pregnant SLE patients.
- First-trimester HCQ levels were not associated with adverse obstetric or fetal outcomes.
- This study supports HCQ monitoring in pregnant SLE patients, as a predictor of severe maternal disease activity.

## Introduction

Systemic lupus erythematosus (SLE) is a multiorgan autoimmune disease that mainly affects women of reproductive age [1]. Pregnancy in such patients carries a higher risk of maternal and fetal adverse outcomes. Pregnant patients with SLE are at risk of disease exacerbation, with rates of flares ranging from 14.7% to 33% in different reports [2–4]*.* Other risks are adverse pregnancy outcomes (APOs) including neonatal lupus syndrome (NLS), preterm delivery due to placental insufficiency, and small-for-gestational-age (SGA) neonates [2, 4, 5]. The presence of lupus anticoagulant (LA), antihypertensive drug use, a physician global assessment (PGA) score >1, a low platelet count, and damage accrual due to SLE at conception (evaluated by SLICC damage index) are predictive of APOs [2, 4], while hypocomplementemia in the first trimester has been associated with the occurrence of maternal flares during pregnancy [4, 6].

Hydroxychloroquine (HCQ) remains the standard reference treatment for SLE: it reduces disease flares [5, 7, 8], including during pregnancy [9]. Sustained use of HCQ during pregnancy may also decrease the risk of NLS recurrence [10]. Therefore, HCQ, which is considered safe during pregnancy [11], is currently recommended for treating SLE patients during this period: by the European League Against Rheumatism (EULAR) since 2016 [1] and by the American College of Rheumatology (ACR) guidelines since 2020 [12].

HCQ levels can be measured, and low blood HCQ levels are associated with SLE disease activity and predict disease exacerbation among the non-pregnant population [13–15]. Additionally, very low or undetectable HCQ levels are an objective marker of severe non-adherence to treatment [14–20]. Very few studies have addressed the interest of HCQ levels in pregnant patients [21, 22]. *Balevicet al.* prospectively studied serum HCQ levels in 50 pregnant patients with rheumatic diseases, including 28 diagnosed with SLE, and unexpectedly found that both higher (>500 ng/ml) and lower average HCQ levels (≤100 ng/ml) during pregnancy (corresponding roughly to 1000 ng/ml and 200 ng/ml in whole blood [23]) were associated with preterm birth and lower neonatal gestational age at birth in SLE patients [22].

Here, we aimed to assess HCQ blood levels during the first trimester of pregnancy as a potential predictor of adverse maternal and obstetric/fetal outcomes and to assess the impact of severe non-adherence in a large cohort of pregnant women with SLE included in the French prospective study of pregnancy and rare diseases (the GR2 study, clinicaltrial.gov NCT02450396).

## Methods

### Patients

We included patients from the ‘Groupe de recherche sur la Grossesse et les Maladies Rares’ (GR2) study (Supplementary Data S1, available at *Rheumatology* online), an ongoing French multicentre prospective observational study of pregnant women with rare auto-immune and/or rheumatological diseases, including SLE and antiphospholipid syndrome (APS), conducted since October 2014 in 76 active centres in France, and previously described [4, 24, 25]. Pregnant women are included by their treating physicians (mainly internists and rheumatologists), who make all treatment decisions in collaboration with obstetricians, and are followed up for 12 months postpartum.

The GR2 study is part of the European network of pregnancy registers in Rheumatology (EuNeP) supported by FOREUM (Foundation for Research in Rheumatology) [26]. It complies with EULAR recommendations regarding core data sets for pregnancy registers in rheumatology [27]. All data are prospectively collected in an electronic case report form; they include demographic, clinical, laboratory, and treatment characteristics. The investigators provided written information and obtained oral consent from each woman. Because all patients received standard care (including measurement of HCQ blood levels, which is routine care in France), written informed consent was not required by French law, as for all the non-interventional studies. The local ethics committee (CPP Ile de France VI) approved the study protocol on 29 August 2012. This project adheres to the principles of the Declaration of Helsinki.

### Inclusion criteria

**Figure 1.**
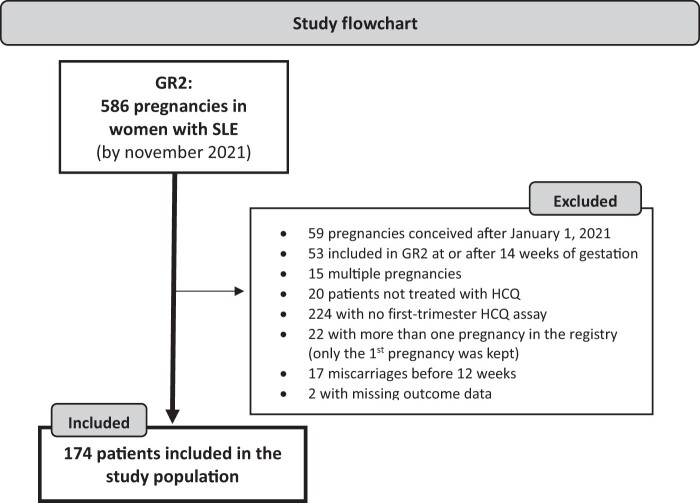
Study flowchart. GR2: Groupe de Recherche sur la Grossesse et les Maladies Rares; HCQ: hydroxychloroquine; SLE: systemic lupus erythematosus

To be eligible for this study, women had to be ≥18 years old, have SLE according to the SLICC 2012 classification criteria [28], have been included in the GR2 study before 14 weeks with a date of conception before 1 January 2021 (Supplementary Data S2, available at *Rheumatology* online), have an ongoing singleton pregnancy that reached at least 12 weeks of gestation, be on a stable daily dose of HCQ until delivery, with at least one determination of HCQ whole-blood level during the first trimester of pregnancy. Only the first singleton pregnancy per woman, and patients with full outcome data were analysed (see Study Flowchart in Fig. 1).

### HCQ measurement and definitions

Available HCQ blood levels during the first trimester of pregnancy were prospectively recorded in the case report form. They were measured from whole-blood samples, by high-performance liquid chromatography, as previously described [13, 29], given that whole blood has proved to be better than serum for assessing the pharmacokinetic/pharmacodynamic relations of HCQ [23]. Of note, serum blood levels are roughly half of whole-blood levels in a given patient [23]. In patients with more than one determination during the first trimester of pregnancy, a mean HCQ level was calculated.

As several cut-offs of interest for HCQ blood levels have been published, we analysed multiple thresholds: 1000 ng/ml [13], 750 ng/ml [30, 31] and 500 ng/ml [15] for HCQ blood level as a therapeutic target, and a threshold of ≤200 ng/ml [18, 23, 32] to assess severe non-adherence.

### Definition of outcomes

Primary outcomes were the occurrence of at least one maternal flare during the second or third trimester of pregnancy and the occurrence of APOs.

Maternal flares were defined according to the SELENA-SLEDAI Flare Index (SFI) [33]. This composite score captures an assessment of new or worsening disease activity (measured by the SELENA-SLEDAI score), any increase in the PGA of disease activity (on a VAS or visual analogue scale: 0–3 mm), and new or worsening disease activity, medication changes and hospitalizations for SLE flares not captured with the use of the SLEDAI. It classifies flares as mild/moderate or severe.

To assess obstetrical and fetal outcomes, we defined APOs [2] as a composite binary variable (the occurrence of at least one of the following events *vs* the non-occurrence of any of them): an otherwise unexplained intrauterine fetal death (IUFD) ≥12 weeks, a neonatal death (within 28 days after birth), placental insufficiency (fetal growth restriction, preeclampsia/eclampsia, HELLP syndrome and/or placental abruption) leading to preterm delivery <37 weeks, and/or SGA birth weight (below the third percentile according to the French AUDIPOG curve [34]). We also assessed these complications individually.

### Statistical analyses

Continuous variables were expressed as means and standard deviations (SD) if normally distributed, or as medians and interquartile ranges (IQR) if not normally distributed. Categorical variables were expressed as percentages with their 95% confidence interval (95% CI).

Continuous variables were compared with the Student’s *t* test or Wilcoxon’s rank-sum test according to the distribution of data (parametric and non-parametric variables, respectively), while categorical variables were compared with χ^2^ test or Fisher’s exact test, according to the sample sizes. Statistical significance was defined as *P* ≤ 0.05. Logistic regression analyses were performed to identify factors associated with maternal flares and APOs.

All statistical analyses were conducted with STATA v.13.0 (StataCorp, College Station, TX USA).

## Results

### Patient characteristics at enrolment

This study included 174 pregnant women with SLE from 20 French centres (see Fig. 1 and Supplementary Table S1, available at *Rheumatology* online). Median age was 32.1 years (IQR 28.8–35.2), and median disease duration was 8.3 years (IQR 4.9–13.8). Tables 1 and 2 detail their baseline characteristics.

**Table 1.** Comparison of baseline characteristics in patients with and without maternal flares in the second and third trimesters of gestation

	Total population	Flare	No flare	*P* value
	(*n* = 174)	(*n* = 30)	(*n* = 144)	
Maternal characteristics				
Age at pregnancy (years), median (IQR)	32.1 (28.8–35.2)	33.6 (29.3–36.3)	31.8 (28.7–34.9)	0.20
Family geographical origin				
European descent	125 (71.8%)	23 (76.7%)	102 (70.8%)	0.52
African descent	26 (14.9%)	5 (16.7%)	21 (14.6%)	0.78
Asian descent	15 (8.6%)	2 (6.7%)	13 (9.0%)	1
Other	8 (4.6%)	0 (0.00%)	8 (5.6%)	0.35
Overweight (BMI ≥ 25 kg/m^2^)	50 (28.7%)	5 (16.7%)	45 (31.2%)	0.12
Professional activity	138 (79.3%)	26 (86.7%)	112 (77.8%)	0.27
Couple life	166 (95.4%)	29 (96.7%)	137 (95.1%)	1
Active smokers	16 (9.2%)	5 (16.7%)	11 (7.6%)	0.16
Active alcohol consumption	2 (1.1%)	1 (3.3%)	1 (0.7%)	0.32
Disease characteristics at inclusion				
Disease duration (years), median (IQR)	8.3 (4.9–13.8)	9.3 (4.9–14.1)	7.9 (5–13.6)	0.69
Associated APS	23 (13.2%)	1 (3.3%)	22 (15.3%)	0.13
Clinical phenotype (*n* = 23)				
Obstetric	10/23 (43.5%)	1 (100%)	9 (40.9%)	0.43
Thrombotic	16/23 (69.6%)	1 (100%)	15 (68.2%)	1
Previous renal involvement	59 (33.9%)	6 (20.0%)	53 (36.8%)	0.08
Total SLEPDAI, median (IQR)	0 (0–2)	2 (0–2)	0 (0–2)	0.18
PGA of disease activity (VAS: 0–3 mm), median (IQR)^a^	0.1 (0.0–0.2)	0.1 (0.1–0.4)	0.1 (0–0.2)	0.11
Complete remission (DORIA/Zen definition)	55 (31.6%)	7 (23.3%)	48 (33.3%)	0.28
Remission (DORIS definition) – (*n* = 172)^a^	116 (67.4%)	**15 (51.7%)**	**101 (70.6%)**	**0.05**
LLDAS (*n* = 172)^a^	136 (79.1%)	20 (68.9%)	116 (81.1%)	0.14
SLICC/ACR Damage Index ≥1 (*n* = 171)^b^	25 (14.6%)	4 (13.3%)	21 (14.9%)	1
Laboratory characteristics during SLE history				
Low platelets (<100 000/mm^3^)	39 (22.4%)	7 (23.3%)	32 (22.2%)	0.89
Anti-dsDNA antibodies	150 (86.2%)	28 (93.3%)	122 (84.7%)	0.38
First-trimester hypocomplementemia (*n* = 170)^c^	37 (21.8%)	7 (23.3%)	30 (21.4%)	0.82
At least one positive aPL	44 (25.3%)	7 (23.3%)	37 (25.7%)	0.79
LA	31 (17.8%)	6 (20.0%)	25 (17.4%)	0.73
IgG/IgM aCL	29 (16.7%)	4 (13.3%)	25 (17.4%)	0.79
IgG/IgM anti-β2GPI	16 (9.2%)	2 (6.7%)	14 (9.7%)	1
Triple positive aPL	13 (7.5%)	2 (6.7%)	11 (7.6%)	1
Treatments				
HCQ daily dose				
400 mg/day	148 (85.1%)	24 (80.0%)	124 (86.1%)	0.39
200 mg/day	12 (6.9%)	4 (13.3%)	8 (5.6%)	0.13
A different dose	14 (8.0%)	2 (6.7%)	12 (8.3%)	1
First-trimester HCQ blood concentration (ng/mL), median (IQR)	855 (564–1229)	806 (572–1118)	886 (559–1246)	0.43
HCQ levels >1000 ng/mL	68 (39.1%)	10 (33.3%)	58 (40.3%)	0.48
HCQ levels >750 ng/mL	106 (60.9%)	16 (53.3%)	90 (62.5%)	0.35
HCQ levels >500 ng/mL	140 (80.5%)	25 (83.3%)	115 (79.9%)	0.66
HCQ levels ≤200 ng/mL	15 (8.6%)	3 (10.0%)	12 (8.3%)	0.73
HCQ levels ≤100 ng/mL	11 (6.3%)	1 (3.3%)	10 (6.9%)	0.69
Glucocorticoids	84 (48.3%)	16 (53.3%)	68 (47.2%)	0.54
Prednisone equivalent dose (mg/day), median (IQR) (*n* = 84)	5 (5–8.5)	**7.50 (5–10)**	**5 (5–7.5)**	**0.05**
Immunosuppressants^d^	45 (25.9%)	9 (30.0%)	36 (25.0%)	0.57
Low-molecular-weight heparin	54 (31.0%)	7 (23.3%)	47 (32.6%)	0.32
Aspirin	135 (77.6%)	21 (70.0%)	114 (79.2%)	0.27
Combined aspirin and low-molecular-weight heparin	52 (29.9%)	7 (23.3%)	45 (31.2%)	0.39
Antihypertensives	20 (11.5%)	3 (10.0%)	17 (11.8%)	1

Results are indicated as number (percentage), unless indicated differently. Values in bold indicate significant associations.

a PGA of disease activity unavailable for two patients, preventing the calculation of states of remission (DORIS) and LLDAS in those patients.

b SLICC/ACR Damage Index unavailable for three patients.

c First-trimester complementemia unavailable for four patients.

d Immunosuppressants: azathioprine (*n* = 37, 21.3%) and tacrolimus (*n* = 9, 5.2%); two women received both.

aCL: anticardiolipin antibodies; anti-β2GPI: Anti-Beta 2 Glycoprotein I antibodies; anti-dsDNA: anti double stranded DNA antibody; aPL: antiphospholipid antibodies; APS: antiphospholipid syndrome; BMI: body mass index; HCQ: hydroxychloroquine; IQR: interquartile range; LA: lupus anticoagulant; LLDAS: lupus low disease activity state; PGA: Physician Global Assessment; SLEPDAI: Systemic Lupus Erythematosus Pregnancy Disease Activity Index; SLICC/ACR: Systemic Lupus International Collaborating Clinics/American College of Rheumatology; VAS: visual analogue scale.

**Table 2.** Comparison of baseline characteristics in patients who did and did not develop adverse pregnancy outcomes (APO)

	Total population	APO	No APO	*P* value
	(*n* = 174)	(*n*=28)	(*n*=146)	
Maternal characteristics				
Age at pregnancy (years), median (IQR)	32.1 (28.8–35.2)	32.8 (29.2–35.7)	31.9 (28.7–34.9)	0.60
Family geographical origin				
European descent	125 (71.8%)	19 (67.9%)	106 (72.6%)	0.61
African descent	26 (14.9%)	7 (25.0%)	19 (13.0%)	0.14
Asian descent	15 (8.6%)	1 (3.6%)	14 (9.6%)	0.47
Others	8 (4.6%)	1 (3.6%)	7 (4.8%)	1
Overweight (BMI≥25 kg/m^2^)	50 (28.7%)	12 (42.9%)	38 (26.0%)	0.07
Professional activity	138 (79.3%)	19 (67.9%)	119 (81.5%)	0.10
Couple life	166 (95.4%)	26 (92.9%)	140 (95.9%)	0.62
Active smokers	16 (9.2%)	**6 (21.4%)**	**10 (6.8%)**	**0.01**
Active alcohol consumption	2 (1.1%)	0 (0.00%)	2 (1.4%)	1
Disease characteristics at inclusion				
Disease duration (years), median (IQR)	8.3 (4.9–13.8)	**12.9 (6.0–18.3)**	**7.8 (4.8–12.9)**	**0.04**
Associated APS	23 (13.2%)	**8 (28.6%)**	**15 (10.3%)**	**0.01**
Clinical phenotype (*n*=23)				
Obstetric	10/23 (43.5%)	2 (25.0%)	8 (53.3%)	0.38
Thrombotic	16/23 (69.6%)	7 (87.5%)	9 (60.0%)	0.34
Previous renal involvement	59 (33.9%)	**15 (53.6%)**	**44 (30.1%)**	**0.02**
Total SLEPDAI, median (IQR)	0 (0–2)	2 (0–2.5)	0 (0–2)	0.20
PGA of disease activity (VAS: 0–3 mm), median (IQR)^a^	0.1 (0.0–0.2)	0.1 (0.0–0.3)	0.1 (0.0–0.2)	0.77
Complete remission (DORIA/Zen definition)	55 (31.6%)	7 (25.0%)	48 (32.9%)	0.41
Remission (DORIS definition) – (*n*=172)^a^	116 (67.4%)	15 (55.6%)	101 (69.7%)	0.15
LLDAS (*n*=172)^§^	136 (79.1%)	20 (74.1%)	116 (80.0%)	0.49
SLICC/ACR Damage Index ≥1 (*n*=171)^b^	25 (14.6%)	**9 (33.3%)**	**16 (11.1%)**	**0.003**
Laboratory characteristics during SLE history				
Low platelets (<100 000/mm^3^)	39 (22.4%)	9 (32.1%)	30 (20.5%)	0.18
Anti-dsDNA antibodies	150 (86.2%)	26 (92.9%)	124 (84.9%)	0.37
First-trimester hypocomplementemia (*n*=170)^c^	37 (21.8%)	8 (28.6%)	29 (20.4%)	0.34
At least one positive aPL	44 (25.3%)	**17 (60.7%)**	**27 (18.5%)**	**<0.001**
LA	31 (17.8%)	**13 (46.4%)**	**18 (12.3%)**	**<0.001**
IgG/IgM aCL	29 (16.7%)	**10 (35.7%)**	**19 (13.0%)**	**0.01**
IgG/IgM anti-β2GPI	16 (9.2%)	**6 (21.4%)**	**10 (6.8%)**	**0.03**
Triple positive aPL	13 (7.5%)	**6 (21.4%)**	**7 (4.8%)**	**0.008**
Treatments				
HCQ daily dose				
400 mg/day	148 (85.1%)	23 (82.1%)	125 (85.6%)	0.57
200 mg/day	12 (6.9%)	2 (7.1%)	10 (6.8%)	1
A different dose	14 (8.0%)	3 (10.7%)	11 (7.5%)	0.70
First-trimester HCQ blood concentration (ng/mL), median (IQR)	855 (564–1229)	812 (429–1227)	864 (574–1229)	0.56
HCQ levels >1000 ng/mL	68 (39.1%)	9 (32.1%)	59 (40.4%)	0.41
HCQ levels >750 ng/mL	106 (60.9%)	15 (53.6%)	91 (62.3%)	0.38
HCQ levels >500 ng/mL	140 (80.5%)	20 (71.4%)	120 (82.2%)	0.19
HCQ levels ≤200 ng/mL	15 (8.6%)	3 (10.7%)	12 (8.2%)	0.71
HCQ levels ≤100 ng/mL	11 (6.3%)	2 (7.1%)	9 (6.2%)	0.69
Glucocorticoids	84 (48.3%)	**19 (67.9%)**	**65 (44.5%)**	**0.02**
Prednisone equivalent dose (mg/day), median (IQR) (*n*=84)	5 (5–8.5)	6 (5–10)	5 (5–8)	0.47
Immunosuppressants^d^	45 (25.9%)	11 (39.3%)	34 (23.3%)	0.08
Low-molecular-weight heparin	54 (31.0%)	**15 (53.6%)**	**39 (26.7%)**	**0.005**
Aspirin	135 (77.6%)	**28 (100%)**	**107 (73.3%)**	**0.001**
Combined aspirin and low-molecular-weight heparin	52 (29.9%)	**16 (57.1%)**	**36 (24.7%)**	**0.001**
Antihypertensives	20 (11.5%)	**11 (39.3%)**	**9 (6.2%)**	**<0.001**

Results are indicated as number (percentage), unless indicated differently. Values in bold indicate significant associations.

a PGA of disease activity unavailable for two patients, preventing the calculation of states of remission (DORIS) and LLDAS in those patients.

b SLICC/ACR Damage Index unavailable for three patients.

c First-trimester complementemia unavailable for four patients.

d Immunosuppressants: azathioprine (*n* = 37, 21.3%) and tacrolimus (*n* = 9, 5.2%); two women received both.

aCL: anticardiolipin antibodies; anti-β2GPI: Anti-Beta 2 Glycoprotein I antibodies; anti-dsDNA: anti double stranded DNA antibody; aPL: antiphospholipid antibodies; APS: antiphospholipid syndrome; BMI: body mass index; HCQ: hydroxychloroquine; IQR: interquartile range; LA: lupus anticoagulant; LLDAS: lupus low disease activity state; PGA: Physician Global Assessment; SLEPDAI: Systemic Lupus Erythematosus Pregnancy Disease Activity Index; SLICC/ACR: Systemic Lupus International Collaborating Clinics/American College of Rheumatology; VAS: visual analogue scale.

The HCQ daily dose was 400 mg/day for 148 patients (85.1%), 200 mg/day for 12 (6.9%), while 14 patients (8.0%) received a different dose. Patients had a median HCQ whole-blood level during the first trimester of 855 ng/ml (IQR 564–1229). Severe vomiting (*hyperemesis gravidarum*) during the first trimester interfered with the medication intake of 21 patients (12.1%), but there were no statistically significant differences in HCQ blood levels between patients with and without vomiting (median HCQ blood level 740 ng/ml, IQR 528–1240 *vs* 870 ng/ml, IQR 569–1219, respectively; *P* *=* 0.26). Thirty-four patients (19.5%) had subtherapeutic HCQ levels ≤500 ng/ml; 15 (8.6%) of them were classified as severely non-adherent with HCQ levels ≤200 ng/ml.

### Maternal flares

Thirty patients (17.2%, 95% CI 12.3–23.7) had at least one flare during the second or third trimester of pregnancy. Flares were severe for four of them (2.3%, 95% CI 0.8–6.0): two haematological flares (immune thrombocytopenia and Evans syndrome), one flare with serosal and cutaneous manifestations, and one with renal, serosal and articular involvement. All four women required a corticoid dose increase and/or the addition of an immunosuppressive drug to the background treatment, and three also required hospitalization. All four had live births, two of them preterm (birth at 25 and 28 weeks of gestation), due to early preeclampsia.

Comparison of baseline characteristics in patients with and without maternal flares are reported in Table 1. Patients with flares during pregnancy had been less frequently in remission (according to the DORIS definition) at inclusion (51.7% *vs* 70.6%, *P* *=* 0.05).

**Figure 2.**
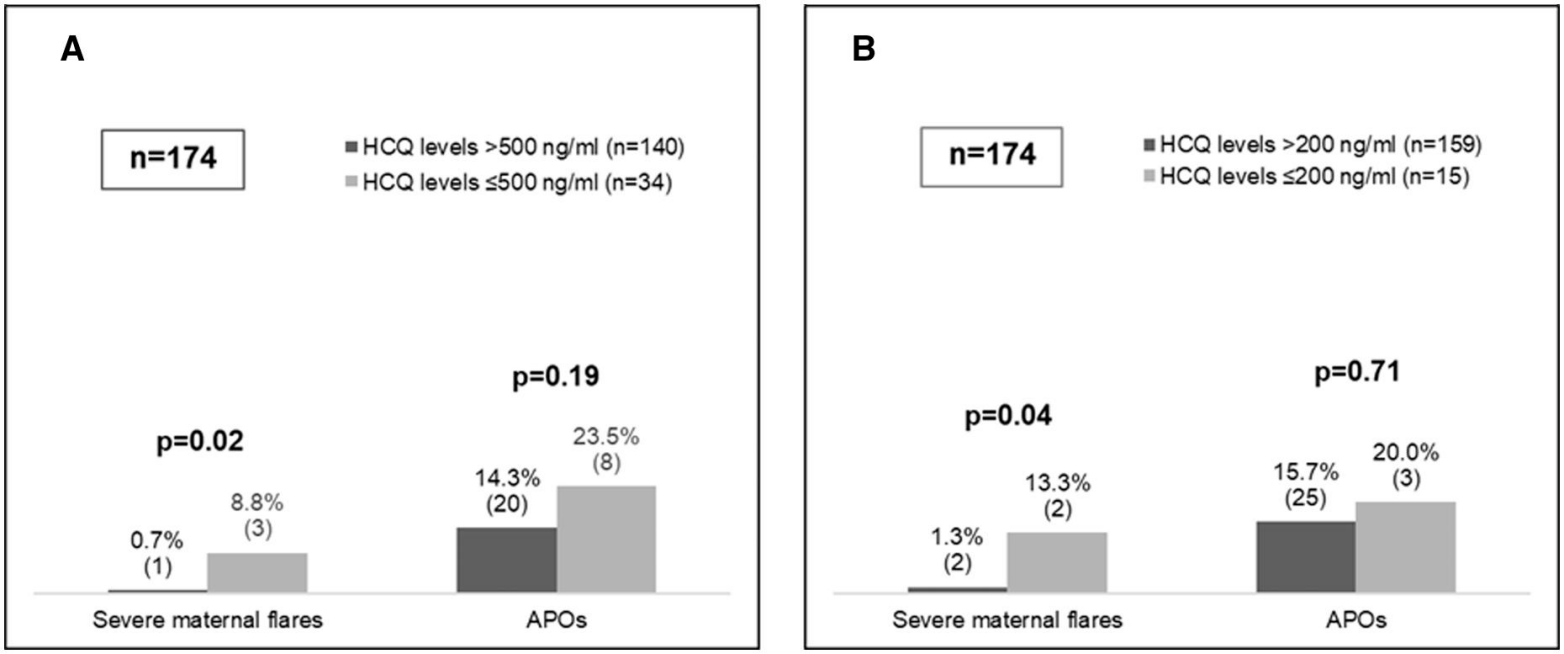
Severe maternal flares and APOs according to different thresholds for HCQ blood levels. (**A**) Therapeutic threshold (therapeutic >500 ng/mL; subtherapeutic ≤500 ng/mL). (**B**) Adherence threshold (adherence >200 ng/mL; non-adherence ≤200 ng/mL). APOs: adverse pregnancy outcomes; HCQ: hydroxychloroquine

Overall maternal flares (regardless of the severity) did not differ significantly by HCQ levels. Nonetheless, patients with subtherapeutic HCQ levels ≤500 ng/ml had significantly more severe flares than those with a level >500 ng/ml (8.8% *vs* 0.7%, *P* *=* 0.02) (Fig. 2 and Table 3). Similarly, patients with non-adherent HCQ levels (≤200 ng/ml) had significantly more severe flares than the subgroup with HCQ levels >200 ng/ml (13.3% *vs* 1.3%, *P* *=* 0.04) (Fig. 2 and Table 4). An additional subanalysis comparing patients with therapeutic (>500 ng/ml) and non-adherent HCQ levels (≤200 ng/ml) also showed that severe flares were significantly more frequent in patients with non-adherent levels (Supplementary Table S2, available at *Rheumatology* online). Comparisons using the other tested HCQ thresholds (1000 ng/ml and 750 ng/ml) did not show significant differences (see Supplementary Table S3, available at *Rheumatology* online).

**Table 3.** Maternal, obstetrical and fetal outcomes in the whole cohort, and in subgroups by HCQ blood levels (therapeutic >500 ng/mL; subtherapeutic ≤500 ng/mL)

	Total population	HCQ levels >500 ng/mL	HCQ levels ≤500 ng/mL	*P* value (>500 vs ≤500)
	(*n* = 174)	(*n* = 140)	(*n* = 34)	
Maternal disease activity during the 2nd and 3rd trimesters of pregnancy				
Maternal flares:				
At least 1 flare during the 2nd or 3rd trimester	30 (17.2%)	25 (17.9%)	5 (14.7%)	0.80
At least 1 severe flare during the 2nd or 3rd trimester	4 (2.3%)	**1 (0.7%)**	**3 (8.8%)**	**0.02**
Obstetrical and fetal complications				
Adverse pregnancy outcomes (APOs):	28 (16.1%)	20 (14.3%)	8 (23.5%)	0.19
Placental insufficiency (FGR, preeclampsia/eclampsia, HELLP, placental abruption) leading to preterm delivery <37 weeks	19 (10.9%)	14 (10.0%)	5 (14.7%)	0.54
Neonatal death (within 28 days after birth)	1 (0.6%)	1 (0.7%)	0 (0.0%)	1
Unexplained IUFD ≥12 weeks	3 (1.7%)	2 (1.4%)	1 (2.9%)	0.48
Small-for-gestational-age birth weight	7 (4.0%)	4 (2.9%)	3 (8.8%)	0.14
Gestational age at delivery, median (IQR)	38 (36–39)	38 (36–39)	38 (37–39)	0.74
Preterm delivery (<37 weeks) (*n* = 169 live births)	39 (23.1%)	33 (24.3%)	6 (18.2%)	0.46

Results are indicated as number (percentage), unless indicated differently. Values in bold indicate significant associations.

FGR: fetal growth restriction; HCQ: hydroxychloroquine; HELLP: Hemolysis, Elevated Liver enzymes and Low Platelets syndrome; IQR: interquartile range; IUFD: intrauterine fetal death.

**Table 4.** Maternal, obstetrical and fetal outcomes in the whole cohort, and subgroups by adherent HCQ blood levels (adherence >200 ng/mL; non-adherence ≤200 ng/mL)

	Total population	HCQ levels >200 ng/mL	HCQ levels ≤200 ng/mL	*P* value (>200 vs ≤200)
	(*n* = 174)	(*n* = 159)	(*n* = 15)	
Maternal disease activity during the 2nd and 3rd trimesters of pregnancy				
Maternal flares:				
At least 1 flare during the 2nd or 3rd trimester	30 (17.2%)	27 (17.0%)	3 (20.0%)	0.73
At least 1 severe flare during the 2nd or 3rd trimester	4 (2.3%)	**2 (1.3%)**	**2 (13.3%)**	**0.04**
Obstetrical and fetal complications				
Adverse pregnancy outcomes:	28 (16.1%)	25 (15.7%)	3 (20.0%)	0.71
Placental insufficiency (FGR, preeclampsia/eclampsia, HELLP, placental abruption) leading to preterm delivery <37 weeks	19 (10.9%)	17 (10.7%)	2 (13.3%)	0.67
Neonatal death (within 28 days after birth)	1 (0.6%)	1 (0.6%)	0 (0.0%)	1
Unexplained IUFD ≥12 weeks	3 (1.7%)	3 (1.9%)	0 (0.0%)	1
Small-for-gestational-age birth weight	7 (4.0%)	6 (3.8%)	1 (6.7%)	0.47
Gestational age at delivery, median (IQR)	38 (36–39)	38 (36–39)	38 (37–39)	0.97
Preterm delivery (<37 weeks) (*n* = 169 live births)	39 (23.1%)	36 (23.4%)	3 (20.0%)	1

Results are indicated as number (percentage), unless indicated differently. Values in bold indicate significant associations.

FGR: fetal growth restriction; HCQ: hydroxychloroquine; HELLP: Hemolysis, Elevated Liver enzymes and Low Platelets syndrome; IQR: interquartile range; IUFD: intrauterine fetal death.

On univariate analyses, first-trimester HCQ levels were not associated with overall maternal flares (OR 0.99; 95% CI 0.99–1.00; *P* *=* 0.39), but higher first-trimester HCQ levels were associated to less severe flares (OR 0.997; 95% CI 0.995–0.999; *P* *=* 0.03). Multivariate analyses were not performed due to the small number of severe flares.

### Obstetric and fetal outcomes

Live births occurred among 169 patients (97.1%), at a median gestational age of 38 weeks (IQR 36–39). Twenty-eight patients (16.1%, 95% CI 11.3–22.4) had at least one APO. These included 19 (10.9%) preterm births due to placental insufficiency, seven (4.0%) SGA infants, three (1.7%) IUFD ≥12 weeks, and one (0.6%) neonatal death (of note, two additional pregnancies were terminated due to chromosomal abnormalities and were not included in the APOs).

Table 2 compares baseline characteristics in patients with and without APOs. LA-positivity (46.4% *vs* 12.3%, *P* <0.001), at least one antiphospholipid antibody (aPL) (60.7% *vs* 18.5%, *P* <0.001), an associated APS (28.6% *vs* 10.3%, *P* *=* 0.01), and greater damage accrual (33.3% *vs* 11.1%, *P* *=* 0.003) were all significantly more frequent among patients with an APO.

APOs did not differ significantly by HCQ level, regardless of the threshold. This was the case both for subtherapeutic HCQ levels (≤500 ng/ml *vs* >500 ng/ml: 23.5% *vs* 14.3%, *P* *=* 0.19) (Fig. 2 and Table 3) and non-adherent HCQ levels (≤200 ng/ml *vs* >200 ng/ml: 20.0% *vs* 15.7%, *P* *=* 0.71) (Fig. 2 and Table 4). Comparisons for the other HCQ thresholds (1000 ng/ml and 750 ng/ml) did not show significant results (see Supplementary Table S3, available at *Rheumatology* online).

## Discussion

This large prospective study of pregnant SLE patients assessed HCQ whole-blood levels in this population for the first time and found that subtherapeutic (≤500 ng/ml) and non-adherent HCQ levels (≤200 ng/ml) were associated with severe maternal flares during pregnancy, but not with obstetric or fetal outcomes.

Overall SLE flares were not significantly associated with HCQ levels, but severe maternal flares were associated with both ≤500 ng/ml and ≤200 ng/ml cut-offs (corresponding to subtherapeutic and severe non-adherence levels, respectively). HCQ use during pregnancy prevented SLE flares, as *Clowseet al.* established in a meta-analysis of datasets from seven lupus pregnancy cohorts [35]. Consistently with this finding, a retrospective study showed that discontinuation of HCQ during pregnancy was associated with more disease flares [36]. Several studies [13, 14, 37] also show that low blood levels of HCQ are related to increased disease activity outside pregnancy (of note, since there is no clear consensus about the optimal HCQ threshold for assessing therapeutic and adherent levels, we tested several thresholds). However, except for a relatively recent small study [22], no data on HCQ levels in pregnant patients with SLE have been available, even though the physiologic changes inherent in pregnancy might well modify its metabolism. *Balevic et al.* studied 28 pregnant women with SLE and found a significant association between average serum HCQ levels during pregnancy and disease activity, with the highest disease activity observed when drug levels were ≤100 ng/ml [22], corresponding roughly to 200 ng/ml in whole blood [23].

These results are consistent with ours, given that a serum HCQ cut-off ≤100 ng/ml corresponds to a whole-blood HCQ cut-off ≤200 ng/ml according to previous studies [23]. However, subtherapeutic HCQ levels (≤500 ng/ml) were still associated with severe flares in our study, while they were protective in that of *Balevic et al.* [22], which is difficult to explain. These discrepancies demonstrate the need for more studies to assess the utility of HCQ drug concentrations in SLE pregnancies and their relations to maternal outcomes.

While we confirmed our previous results about the standard predictor factors of APOs, including LA and damage, we found no association between HCQ levels and APOs. This contrasts with the results by *Balevicet al.* described above. Their study of 28 women with a SLE diagnosis found that both higher (>500 ng/ml) and lower serum HCQ levels (≤100 ng/ml)—corresponding roughly to 1000 ng/ml and 200 ng/ml in whole blood [23]—were associated with preterm birth and lower neonatal gestational age [22]. As these authors acknowledged, this unexpected result should be interpreted carefully in view of the small sample size. Our failure to confirm any impact of HCQ levels on APOs in our larger cohort of pregnant SLE patients is consistent with findings that HCQ use throughout pregnancy (*vs* no use) has no impact on APOs such as fetal loss, preterm delivery or preeclampsia in SLE patients [35, 38, 39].

Finally, non-adherent HCQ levels (≤200 ng/ml) were found in <10% of the cohort, which is in the low range of severe non-adherence found in several studies [17, 40]. This could be explained by the close monitoring of pregnancies in this cohort, and by the widespread monitoring of HCQ levels in clinical practice in France, which may lead to better therapeutic adherence [17].

Our study has some limitations. First, although this is the largest study ever published, the number of patients remains low and prevented us from performing multivariate analyses for severe flares. Second, the precise impact of low HCQ levels in the first trimester could not be assessed because patients had to have an ongoing pregnancy at 12 weeks to be included; some active patients may well have been excluded because their pregnancies ended spontaneously during the first trimester. Also, we only observed the effect of HCQ levels during the first trimester of pregnancy among patients on a stable prescribed dose of HCQ; however, this cannot ensure the therapeutic adherence later in pregnancy, which is another confounding bias. Moreover, few severe flares occurred, possibly because patients were by definition on HCQ therapy, with relatively few severely non-adherent patients. Furthermore, the close monitoring of these pregnancies is demonstrated by the fact that all women had at least one HCQ assay during the first trimester of pregnancy.

In conclusion, first-trimester HCQ whole-blood levels did not predict APOs, but subtherapeutic (≤500 ng/ml) and non-adherent HCQ levels (≤200 ng/ml) were associated with severe maternal flares during pregnancy in this cohort of pregnant SLE patients, mostly with well-controlled SLE. Therefore, this study supports HCQ blood level assessment in pregnant women with SLE, as a predictor of severe maternal disease activity in pregnancy.

## Supplementary material

Supplementary material is available at *Rheumatology* online.

## Supplementary Material

keae302_Supplementary_Data

## Data Availability

Data collected for the study, including deidentified individual participant data and a data dictionary defining each field in the dataset, will be made available to others on request.
