# Supplementary material for: Hydroxychloroquine levels in pregnancy and materno-fetal outcomes in systemic lupus erythematosus patients
Source: Rheumatology (Oxford). 2024 Jun 5;64(3):1225–33. doi: 10.1093/rheumatology/keae302 (PMC11879311; doi:10.1093/rheumatology/keae302)
Supplement: keae302_Supplementary_Data [file keae302_supplementary_data.docx]

**Supplementary materials**

**Supplementary Data S1.** Collaborators of the GR2 group: Noémie Absiror, Emmanuel Azzi, Béatrice Banneville, Antoine Baudet, Constance Beaudouin-Bazire, Cristina Belizna, Rakiba Belkhir, Ygal Benhamou, Emilie Berthoux, Sabine Berthier, Holy Bezanahary, Lisa Biale, Boris Bienvenu, Adrien Bigot, Claire Blanchard-Delaunay, Anne Calas, Pascal Cathebras, Claire Cazalets, Benjamin Chaigne, Olivia Chandesris, Jérémy Chatelais, Emmanuel Chatelus, Fleur Cohen, Pascal Coquerelle, Marion Couderc, Mathilde De Menthon, Claire De Moreuil, Estelle Delattre, Juliette Delforge, Azeddine Dellal, Amélie Denis, Camille Deprouw, Emmanuelle Dernis, Alban Deroux, Sandra Desouches, Philippe Dieudé, Elisabeth Diot, Guillaume Direz, Marine Driessen, Aurélie Du Thanh, Laetitia Dunogeant, Cécile Durant, Isabelle Durieu, Florence Eboue, Marc Fabre, Olivier Fain, Bruno Fautrel, Nicole Ferreira-Maldent, René-Marc Flipo, Aline Frazier, Antoine Froissart, Sophie Georgin-Lavialle, Elisabeth Gervais, Bertrand Godeau, Anne Gompel, Laure Gossec, Phillipe Goupille, Claire Grange, Constance Guillaud-Danis, Aurélie Hummel, Moez Jallouli, Patrick Jego, Stéphanie Jobard, Laurence Josselin-Mahr, Noémie Jourde-Chiche, Anne-Sophie Korganow, Marc Lambert, Delphine Lariviere, Claire Larroche, Augustin Latourte, Christian Lavigne, Thomas Le Gallou, Hervé Levesque, Nicolas Limal, Frédéric Lioté, Valentine Loustau, Emmanuel Maheu, Matthieu Mahevas, Hélène Maillard, Xavier Mariette, Hubert Marotte, Nicolas Martin-Silva, Nihal Martis, Agathe Masseau, François Maurier, Arsène Mekinian, Sara Melboucy-Belkhir, Martin Michaud, Marc Michel, Guillaume Moulis, Jacky Nizard, Jérémy Ora, Rodérau Outh, Elisabeth Pasquier, Jean-Loup Pennaforte, Antoinette Perlat, Hélène Petit Bauer, Evangeline Pillebout, Jean-Maxime Piot, Agnès Portier, Xavier Puechal, Gregory Pugnet, Loic Raffray, Manon Redondin, Alexis Regent, Mélanie Roriz, Gaëtane Sauvêtre, Léa Savey, Nicolas Schleinitz, Raphaele Seror, Aude Servais, Perrine Smets, Vincent Sobanski, Christelle Sordet, Martin Soubrier, Katia Stankovic Stojanovic, Thierry Thomas, Nathalie Tieulé, Marie-Agnès Timsit, Vassilis Tsatsaris, Emmanuelle Weber, Cécile Yelnik.

**Supplementary Data S2.** Dates of inclusion.

We included all pregnancies in the GR2 register from its start (2014) that met this study's inclusion and exclusion criteria. To be able to analyse pregnancy outcomes, we included only pregnancies conceived before January 1, 2021. They therefore had a due date for delivery before October 1, 2021. A date of conception rather than of delivery was chosen to avoid the overrepresentation of pregnancies with preterm delivery, which would have clearly induced bias.

**Supplementary Table S1.** List of 20 French centres with patients included in this study.

| **Name of Centre** | **n** | **%** |
| --- | --- | --- |
| Angers-CHU d'Angers - Hôtel Dieu | 1 | 0.57 |
| Bois-Guillaume-CHU de Rouen - Bois Guillaume | 2 | 1.14 |
| Bordeaux-CHU de Bordeaux - Pellegrin | 2 | 1.14 |
| Grenoble Michallon | 3 | 1.70 |
| Lille-CHRU de Lille - Claude Huriez | 4 | 2.27 |
| Lyon-CH Saint Joseph - Saint Luc | 3 | 1.70 |
| Montivilliers-CH du Havre - Jacques Monod | 1 | 0.57 |
| Nice-CEDEX1-CHU - Pasteur 2 | 1 | 0.57 |
| Nice-CHU de Nice - Archet 1 | 3 | 1.70 |
| Paris-APHP - Bichat | 2 | 1.14 |
| **Paris-APHP - Cochin** | **117** | **67.24** |
| Paris-APHP - Pitié-Salpêtrière | 1 | 0.57 |
| Perpignan-Hôpital Saint Jean | 2 | 1.14 |
| Pessac-CHU de Bordeaux - Haut Lévéque | 14 | 7.95 |
| Poitiers-CHU de Poitiers - Milétrie | 1 | 0.57 |
| Pringy-CHR d'Annecy | 6 | 3.41 |
| Reims-CHU de Reims - Robert Debré | 5 | 2.84 |
| Saint Paul - Hôpital Gabriel Martin | 1 | 0.57 |
| Strasbourg-CHRU de Strasbourg - Civil | 1 | 0.57 |
| Toulouse-CHRU de Toulouse - Purpan | 4 | 2.27 |
| **Total** | **174** | **100** |

**Supplementary Table S2.** Subanalysis of maternal, obstetrical, and fetal outcomes in subgroups by HCQ blood levels (therapeutic >500 ng/mL; non-adherent ≤200 ng/mL).

|  | **HCQ levels >500 ng/mL**  (n=140) | **HCQ levels ≤200 ng/mL**  (n=15) | ***P value***  (>500 vs ≤200) |
| --- | --- | --- | --- |
| ***Maternal disease activity during the 2^nd^ and 3^rd^ trimesters of pregnancy*** | | | |
| **Maternal flares***:*  - At least 1 flare during the 2nd or 3rd trimester  - At least 1 severe flare during the 2nd or 3rd trimester | 25 (17.9%)  **1 (0.7%)** | 3 (20.0%)  **2 (13.3%)** | 0.74  **0.02** |
| ***Obstetrical and fetal complications*** | | | |
| **Adverse pregnancy outcomes:**  - Placental insufficiency (FGR, preeclampsia/eclampsia, HELLP, placental abruption) leading to preterm delivery <37 weeks  - Neonatal death (within 28 days after birth)  - Unexplained IUFD ≥12 weeks  - Small-for-gestational-age birth weight | 20 (14.3%)  14 (10.0%)  1 (0.7%)  2 (1.4%)  4 (2.9%) | 3 (20.0%)  2 (13.3%)  0 (0.0%)  0 (0.0%)  1 (6.7%) | 0.47  0.65  1  1  0.40 |

Results are indicated as number (percentage)

HCQ: hydroxychloroquine; FGR: fetal growth restriction; HELLP: Hemolysis, Elevated Liver enzymes and Low Platelets syndrome, IUFD: intrauterine fetal death.

**Supplementary Table S3.** Maternal, obstetrical and fetal outcomes in the whole cohort, and in subgroups according to other proposed HCQ concentration thresholds (1000 ng/mL and 750 ng/mL, respectively).

|  | **Total population**  (n=174) | **HCQ levels >1000 ng/mL**  (n=68) | **HCQ levels ≤1000 ng/mL**  (n=106) | ***P value***  *(*>1000 vs ≤1000) | **HCQ levels >750 ng/mL**  (n=106) | **HCQ levels ≤750 ng/mL**  (n=68) | ***P value***  (>750 vs ≤750) |
| --- | --- | --- | --- | --- | --- | --- | --- |
| ***Maternal disease activity during the 2nd and 3rd trimesters of pregnancy*** | | | | | | | |
| **Maternal flares***:*  - At least 1 flare during the 2nd or 3rd trimester  - At least 1 severe flare during the 2nd or 3rd trimester | 30 (17.2%)  4 (2.3%) | 10 (14.7%)  0 (0.0%) | 20 (18.9%)  4 (3.8%) | 0.48  0.16 | 16 (15.1%)  1 (0.9%) | 14 (20.6%)  3 (4.4%) | 0.35  0.30 |
| ***Obstetrical and fetal complications*** | | | | | | | |
| **Adverse Pregnancy Outcomes***:*  - Placental insufficiency (FGR, HELLP, placental abruption, preeclampsia/  eclampsia) leading to preterm delivery <37 weeks  - Neonatal death (within 28 days after birth)  - Unexplained IUFD ≥12 weeks  - Small-for-gestational-age birth weight | 28 (16.1%)  19 (10.9%)  1 (0.6%)  3 (1.7%)  7 (4.0%) | 9 (13.2%)  8 (11.8%)  0 (0.0%)  0 (0.0%)  2 (2.9%) | 19 (17.9%)  11 (10.4%)  1 (0.9%)  3 (2.8%)  5 (4.7%) | 0.41  0.77  0.61  0.28  0.71 | 15 (14.1%)  12 (11.3%)  1 (0.9%)  1 (0.9%)  2 (1.9%) | 13 (19.1%)  7 (10.3%)  0 (0.0%)  2 (2.9%)  5 (7.3%) | 0.38  0.83  0.61  0.56  0.11 |

Results are indicated as number (percentage)

HCQ: hydroxychloroquine; FGR: fetal growth restriction; HELLP: Hemolysis, Elevated Liver enzymes and Low Platelets syndrome, IUFD: intrauterine fetal death.
